# Evolutionary directions of single nucleotide substitutions and structural mutations in the chloroplast genomes of the family Calycanthaceae

**DOI:** 10.1186/s12862-020-01661-0

**Published:** 2020-07-31

**Authors:** Wenpan Dong, Chao Xu, Jun Wen, Shiliang Zhou

**Affiliations:** 1 State Key Laboratory of Systematic and Evolutionary Botany, Institute of Botany, Chinese Academy of Sciences, Beijing, 100093 China; 2 Laboratory of Systematic Evolution and Biogeography of Woody Plants, College of Ecology and Nature Conservation, Beijing Forestry University, Beijing, 100083 China; 3 Department of Botany, National Museum of Natural History, Smithsonian Institution, Washington, DC 20013-7012 USA; 4 University of Chinese Academy of Sciences, Beijing, 100049 China

**Keywords:** Chloroplast genome, Calycanthaceae, Structural mutations, Substitution rate, Indels

## Abstract

**Background:**

Chloroplast genome sequence data is very useful in studying/addressing the phylogeny of plants at various taxonomic ranks. However, there are no empirical observations on the patterns, directions, and mutation rates, which are the key topics in chloroplast genome evolution. In this study, we used Calycanthaceae as a model to investigate the evolutionary patterns, directions and rates of both nucleotide substitutions and structural mutations at different taxonomic ranks.

**Results:**

There were 2861 polymorphic nucleotide sites on the five chloroplast genomes, and 98% of polymorphic sites were biallelic. There was a single-nucleotide substitution bias in chloroplast genomes. A → T or T → A (2.84%) and G → C or C → G (3.65%) were found to occur significantly less frequently than the other four transversion mutation types. Synonymous mutations kept balanced pace with nonsynonymous mutations, whereas biased directions appeared between transition and transversion mutations and among transversion mutations. Of the structural mutations, indels and repeats had obvious directions, but microsatellites and inversions were non-directional. Structural mutations increased the single nucleotide mutations rates. The mutation rates per site per year were estimated to be 0.14–0.34 × 10^− 9^ for nucleotide substitution at different taxonomic ranks, 0.64 × 10^− 11^ for indels and 1.0 × 10^− 11^ for repeats.

**Conclusions:**

Our direct counts of chloroplast genome evolution events provide raw data for correctly modeling the evolution of sequence data for phylogenetic inferences.

## Background

Genome evolution is a major theme of biology in the genomics era. The topics cover patterns, directions and rates of substitutions, repeats, rearrangements and recombinations, hybridization and polyploidy, lateral gene transfer, gene families, etc., with varying depth and scope [1, 2]. Genome evolution can be easily demonstrated by genome structure mutations and nucleotide substitutions. The mutations of genome structure include insertions/deletions (indels) and inversions. The nucleotide substitutions are classified into transition (Ts) and transversion (Tv). A genome consists of coding regions and noncoding regions (including introns and intergenic spacers). The nuclear genome is usually very large and complicate. Moreover, plant nuclear genome sequencing is still a bottle-neck due to high costs, bacteria or fungi contaminations, high heterozygosity, etc. [3, 4]. Only economically important or model plants have their complete nuclear genomes sequenced. Instead, the chloroplast genomes unique to plants are very much smaller and easier to manipulate, and it is more likely to give a complete picture of plant genome evolution. Therefore, chloroplast genomes are currently a right choice for genome evolution studies.

Considerable attention has been paid to the evolution rate variations among genes or lineages [5, 6]. The chloroplast genes such as *ndhF*, *matK*, *rbcL* and *trnL-F* have been well studied [7, 8]. Chloroplast DNA shows a biased transition (Ts) mutation toward A and T. For example, the frequency of A and T at the 3rd codon position is fourfold of other nucleotides in the *rbcL* gene of angiosperms [8]. This explains why the chloroplast genomes are usually A/T rich. The rates of the single nucleotide mutations are not uniform among different genes. Transition/transversion ratios (Ts/Tv) are 0.9 for *rbcL* and 1.4 for *matK* [9, 10]. Among the eight transversion mutation possibilities, from A to T and C to G are significantly less frequent than the other four possibilities [11–13]. Whether this observation is a general pattern or only a special case remains to be tested at genome level.

Structural mutations of genomes convey important evolution information of organisms. The indels and inversions are rich in chloroplast genomes and can be reliably identified and used to reveal the evolution of organisms [14, 15]. Structural mutations are not randomly distributed throughout the chloroplast genome [14]. Tandem repeat-induced indels showed a statistically significant bias towards A/T-rich and the indel mutation rate was estimated to be approximately 0.8 ± 0.04 × 10^− 9^ per site per year in Poaceae [16]. Short inversions also have a widespread occurrence in chloroplast genomes and often form stem-loop structures [17–19]. Understanding the evolution of such structural mutations is crucial for making full and correct use of the genome information [20].

**Fig. 1:**
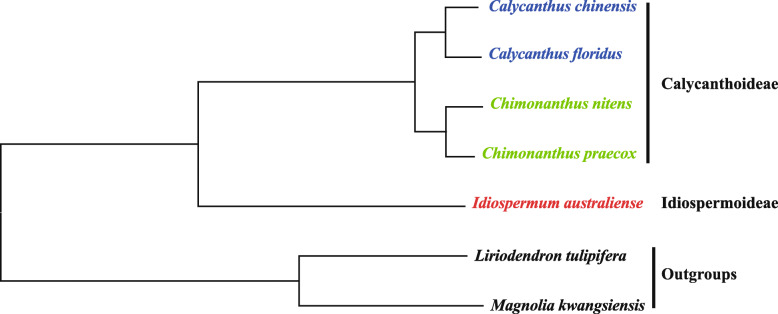
Phylogenetic relationships five species belonging to two subfamilies, three genera of the Calycanthaceae using two species of Magnoliaceae as outgroups

The rates of DNA mutation is one of the core questions in molecular evolution [5]. The mutation rates can be estimated from either mutation accumulation (MA) lines [21, 22] or phylogenetic inference [23]. For the latter method, if the branch age is known, the absolute substitution rate can be calculated. The branch age is usually dated using calibrated molecular clocks. It is a common practice to infer the directions of mutations according to a phylogeny which, unfortunately, is usually based on the same dataset. It would be better if the phylogeny is independent to dataset [24]. The family Calycanthaceae serves as an ideal reference because the phylogenetic relationships within the family are self-evident. Calycanthaceae is a small family holding a position at the base of Laurales [25]. There are in total about 10 species belonging to three genera of two subfamilies (see below for more details). The phylogenetic topology of the family has only one possibility at subfamily, genus and species ranks (Fig. 1), which enables us to infer the chloroplast genome evolution in this family at different taxonomic ranks. In this study, we use Calycanthaceae as a model to empirically observe the directions and to estimate the rates of different mutations imprinted in the chloroplast genomes at different taxonomic ranks. More specifically, we are going to answer (1) if there are significant mutation rate differences between the two subfamilies, between the two genera within subfamily Calycanthoideae, and among species within *Calycanthus* and within *Chimonanthus*; and (2) what the evolutionary patterns, directions of nucleotide substitutions and structural mutations in the Calycanthaceae chloroplast genomes are like. Such kind of empirical data is of values for precisely modeling the chloroplast genome evolution.

## Methods

### The family Calycanthaceae and sampling strategies

Calycanthaceae holds a basal position in Laurales [26]. The family is subdivided into two subfamilies: subfamily Idiospermoideae [one genus, one species, *Idiospermum australiense* (Diels) S. T. Blake] and subfamily Calycanthoideae (two genera, ca. nine species). Subfamily Idiospermoideae was sometimes considered a distinct family [27] but it is more natural in Calycanthaceae. There are three species in *Calycanthus*, one in China (*C. chinensis* Cheng & S. Y. Chang) and two in USA (*C. floridus* L. and *C. occidentalis* Hook. & Arn.). *Calycanthus chinensis* was once separated and put in the monotypic genus *Sinocalycanthus*. There are about six species in *Chimonanthus*. There are deciduous species like *Ch. praecox* L., and evergreen species like *Ch. nitens* Oliv. (see Zhou et al. 2006 for details).

We selected five species, *C. chinensis*, *C. floridus*, *Ch. nitens*, *Ch. praecox* and *Idiospermum australiense*, to represent two subfamilies, three genera and deciduous and evergreen species within *Chimonanthus*. Thus their phylogenetic relationships are intuitively quite clear (Fig. 1). The chloroplast genome of *C. floridus* has been determined [28] and the genomes of other four species need to be determined. Young and healthy leaves of these species were collected. The voucher details of the samples are given in Supplementary Table S1. According to APG IV [29], more basal species *Liriodendron tulipifera* L. and *Magnolia kwangsiensis* Figlar & Noot. are used as outgroups.

### Chloroplast genome sequencing and annotation

Genomic DNA was extracted from the silica gel-dried leaves of four species using mCTAB method [30] and purified using the Wizard DNA Clean-Up System (A7280, Promega). The fragments covering the whole chloroplast genomes were amplified using the universal primers provided by Dong et al. [31]. Specific primers were designed based on the chloroplast genome of *C. floridus* using Primer Premier v. 5.0 (Premier Biosoft International, CA, USA) and Oligo v. 6.71 (Molecular Biology Insights, CO, USA) to bridge the gaps (Supplementary Table S2).

The genomes were assembled using Sequencher ver. 4.7 (Gene Code) with the genome of *C. floridus* as a reference. The resulting genomes were annotated using Dual Organellar Genome Annotator (DOGMA) [32]. All tRNA genes were further verified using the corresponding structures predicted by tRNAscan-SE 1.21 [33].

### Codon usage analysis

Codon usage was determined for all protein-coding genes. The frequency of codon usage and the amino acid composition was determined using MEGA version 7 [34].

### Chloroplast genome alignments and molecular dating

All five Calycanthaceae chloroplast genomes were aligned together with the outgroup *L. tulipifera* and *M. kwangsiensis* using MUSCLE v3.7 [35] and adjusted manually using Se-Al 2.0 [36].

To estimate the divergence times of subfamilies, genera and species within Calycanthaceae, we conducted molecular dating analyses by adding 22 more genome sequences from GenBank (Supplementary Table S3) representing the major monophyletic branches of basal angiosperms, monocots, rosids, Saxifragales, and asterids. Sequences of the eighty-three coding genes were extracted from the genomes, aligned and concatenated into a super matrix. Rare insertions and unreliably aligned regions were excluded from analyses. The dating methods were the same as Xue et.al [37]. For calibration, three constraints were used: (i) the angiosperm crown group was set to a minimum age of 131.8 Mya [38]; (ii) the eudicots crown was set to a minimum age of 125 Mya; (iii) the crown group of Calycanthaceae and Magnoliaceae was set to a maximum age of 140 Mya based on the onset of angiosperm radiation, and the minimum age of Calycanthaceae was set to a minimum age of 90 Mya according to the fossil record of *Jerseyanthus calycanthoides* [39].

### Genome partition and mutation identification

The genome sequence data were subject to a series of grouping to test the variations of mutation rates and directionality: (1) coding regions, introns and intergenic spacers; (2) transition and transversion of coding genes; (3) synonymous (dS) and nonsynonymous (dN) substitution of coding genes; and (4) gene clusters of the same functions. Hierarchical outgroup usage was adopted to infer the directions of changes. At subfamily rank, *L. tulipifera* and *M. kwangsiensis* was used as outgroups; at genus rank within subfamily Calycanthoideae, *I. australiense* was used as an outgroup; and at species rank within *Calycanthus* and *Chimonanthus*, they were used as outgroups reciprocally. The directions of mutations were identified according to the outgroup(s). If a state is the same as the outgroup(s), the state is considered plesiomorphic, and accordingly, the state different from that of outgroup(s) is considered apomorphic.

**Fig. 2:**
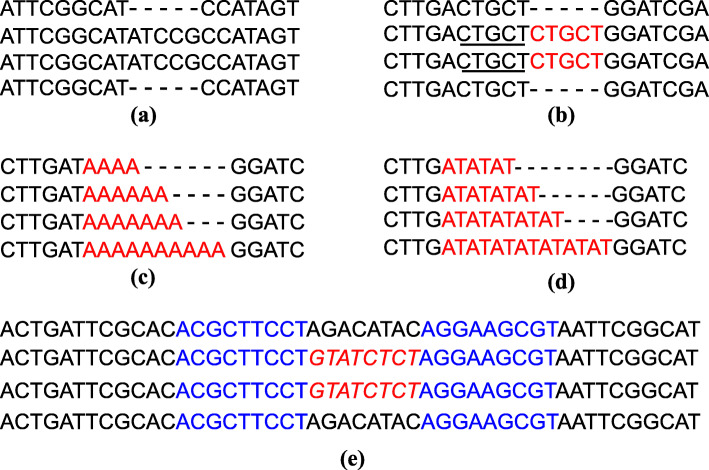
Structural mutation types in chloroplast genomes. **a** indel; **b** repeat; (**c**) and (**d**) microsatellite; **e** inversion

We classified structural mutations into four categories: insertions or deletions (indels, Fig. 2a), repeats (Fig. 2b), microsatellites (Fig. 2c & d), and inversions (Fig. 2e). Although microsatellites are also indels or repeats, we classified them out as a distinct category for their very high variabilities. Inversions were sought out using the REPtuter program [40] first and then confirmed by reexamining the alignments. Each inversion is always accompanied by an inverted repeat at the opposite franking end, which forms a stem-loop structure. Conventional statistics were used to give estimates and to test the significance of the numbers and ratios of the mutations. The Chi-square test, Fisher’s exact test, T-test, significance test of correlation coefficient, and Wilcoxon signed rank test were done with R packages. Genetic distances between any taxa were calculated using MEGA 5 [41].

### Estimation of mutation rate

The rate of mutation per site per year (μ) and its variance (ν) were estimated using the following formula [42].

$$ \upmu =m/(nT),v={\left[v/(nT)\right]}^{1/2} $$

Where *m* is the number of observed mutation, *n* is the number of total sites, and *T* is the divergence time of a node.

The μ and ν values of structural mutations were calculated using the method of Saitou and Ueda [43]. In their method, the total number of structural mutations was divided by the additive time based on branch lengths and by the length of the nucleotide sequences.

### Availability of supporting data

The data sets supporting the results of this article are given as supporting files. All nucleotide sequences were deposited in the NCBI GenBank repository (GenBank accession numbers: MH377056- MH377059).

## Results

### General features of the Calycanthaceae chloroplast genomes

The structures of the four new chloroplast genomes were very similar to that of *C. floridus* (Supplementary Figure S1, Table 1). The genome sizes varied from 153,250 bp (*Ch. praecox*) to 154,746 bp (*I. australiense*). The overall G + C content was 39.23–39.30%, and the coding regions accounted for 58.99 to 59.24%. All five genomes encoded 114 unique genes with identical gene order and gene clusters, 17 or 18 of them were duplicated in the inverted repeat (IR) region (Supplementary Table S4 and Figure S2). Each chloroplast genome comprised four rRNA genes (6S, 23S, 5S, 4.5S), 30 tRNA genes and 80 protein-coding genes including *ycf1*, *ycf2*, *ycf3* and *ycf4*. Each of 16 genes contained a single intron, and each of two genes (*ycf3* and *clpP*) contained two introns. The mRNA gene of *rps12* was generated by transsplicing.

**Table 1:** Major features of the chloroplast genomes of five species in *Calycanthus*, *Chimonanthus* and *Idiospermum*

Genome feature	*C. floridus*	*C. chinensis*	*Ch. nitens*	*Ch. praecox*	*I. australiense*
GenBank accession numbers	AJ428413	MH377059	MH377058	MH377057	MH377056
Size (bp)	153,337	153,346	153,250	153,252	154,767
LSC length (bp)	86,948	86,983	86,882	86,912	85,482
IR length (bp)	23,295	23,284	23,330	23,287	24,860
SSC length (bp)	19,799	19,795	19,708	19,766	19,565
Total number of genes, including *ycf*s	131	131	131	131	132
Number of genes in IR	17	17	17	17	18
Number of genes with introns	18	18	18	18	18
GC content of the genome (%)	39.3	39.27	39.27	39.25	39.23
GC content of protein-coding genes, tRNAs and rRNAs (%)	59.04	59.24	59.07	59.07	58.99

The transition areas between the large single copy region (LSC) and the IR (LSC/IRb and IRa/LSC) were identical in Calycanthoideae but different in Idiospermoideae. In Calycanthoideae, the *rpl2* gene was in the IR region and the *rps19* gene was in LSC region (Supplementary Figure S2). However, in Idiospermoideae, both the *rpl2* and *rps19* genes were in IR region.

The codon usage and amino acid composition frequencies were not significantly different among the five Calycanthaceae chloroplast genomes (Supplementary Figure S3). The total protein coding genes comprised 68,394–68,463 bp that encoded 22,798–22,821 codons. Of these codons, the ATT (3.47%) was the most frequent codon and the TGC (0.30%) was the least. About 10.24% codons encoded leucine, whereas only 1.14% codon encoded cysteine, which were the most and the least frequently used amino acids in the chloroplast genome, respectively.

### Evolution of the single nucleotide polymorphic loci

**Fig. 3:**
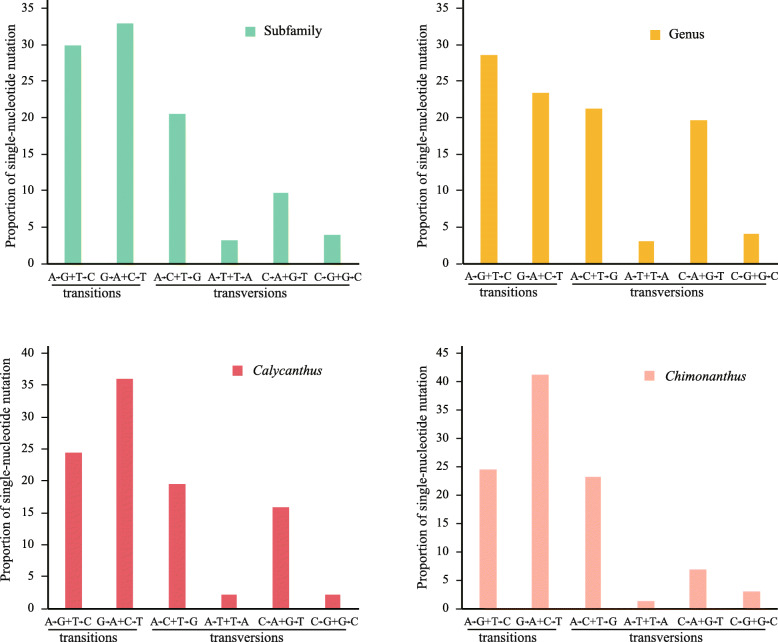
Percentage of each substitution type in the chloroplast genomes of five species belonging to different subfamilies and genera in Calycanthaceae

**Fig. 4:**
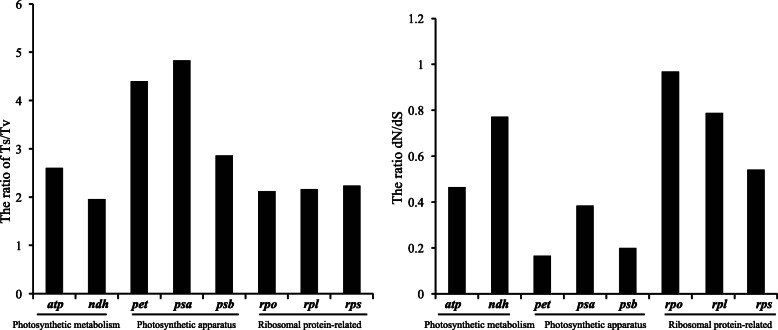
The ratios of Ts/Tv and dN/dS in different functional gene groups

There were 2861 single nucleotide polymorphism (SNP) in the five Calycanthaceae chloroplast genomes. Among them, 2781 were biallelic, 22 (0.79%) SNPs were triallelic, and 58 (2.03%) were parallel mutation SNPs at different taxonomic ranks. The trialletic and parallel mutation SNPs were excluded from subsequent analyses. Of the 2781 sites, the mutation directions of 2573 sites were inferable according to the outgroups. The ratios of Ts to Tv were high at subfamily and species ranks (1.53 and 1.53 or 1.91, respectively) but low at genus rank (1.09), with an overall value of 1.61. The proportions of two transitional mutations and four transversional mutations varied significantly among one another at family, genus and species ranks (G test, *P* < 0.01). Among the four transversion mutations, A → C + T → G mutations were much more common than other three possibilities (Fig. 3). A → T + T → A and G → C + C → G were much fewer. However, the overall ratio of nonsynonymous mutations to synonymous mutations (dN/dS) in the coding regions nearly equaled 1 (Table 2). When the SNPs of the eight functional gene clusters (*atp*, *ndh*, *pet*, *psa*, *psb*, *rpl*, *rpo*, and *rps*) were sorted into photosynthetic metabolism- (*atp* and *ndh*), apparatus- (*pet*, *psa* and *psb*), and ribosomal protein-related (*rpl*, *rpo* and *rps*) groups, there was a tendency that the photosynthetic apparatus-related genes (*pet*, *psa* and *psb*) had the highest Ts/Tv (= 2.86 ~ 4.83) but the lowest dN/dS (= 0.17 ~ 0.38) values (Fig. 4), indicating these genes were under high selection pressure.

**Table 2:** Taxonomic and genomic distribution of the biallelic single nucleotide polymorphic loci in the five chloroplast genomes of Calycanthaceae

		Subfamily		Genus		Species					
Genome region	Length (bp)	Value	%	Value	%	*Calycanthus*	%	*Chimonanthus*	%	Total	%
Total substitutions	130,345	1996	1.53	269	0.21	283	0.22	233	0.18	2781	2.13
Coding region	68,763	980	1.43	118	0.17	116	0.17	118	0.18	1332	1.94
Nonsynonymous	/	489	0.71	53	0.08	61	0.09	64	0.09	667	0.97
Synonymous	/	491	0.71	65	0.09	55	0.08	54	0.08	665	0.97
dN/dS	/	1	/	0.82	/	1.11	/	1.19	/	1	/
Intron	14,104	185	1.31	30	0.21	36	0.26	24	0.17	275	1.95
Intergenic spacer	43,139	831	1.93	121	0.28	131	0.3	91	0.21	1174	2.72

The 2781 biallelic SNPs were subdivided into coding, intron and intergenic spacer regions and sorted into subfamily, genus and species ranks (Table 2). At subfamily rank, there were 1996 SNPs in total: 980 in coding regions, 185 in intron regions, and 831 in intergenic spacer regions. The percentages of SNP to the total lengths were 1.43, 1.31 and 1.93%, respectively, indicating sequences of intergenic spacers were relatively more variable, but not the intron regions. In the coding regions, the ratio of 489 nonsynonymous mutations to 491 synonymous mutations (dN/dS) was very close to 1.0.

The SNPs at genus rank within Calycanthoideae or at species rank within *Calycanthus* and within *Chimonanthus* were much fewer than those at subfamily rank (Table 2). At genus rank, there were 269 mutations, 118 (43.9%) in coding regions, 30 (11.1%) in intron regions, and 121 (45.0%) in intergenic spacer regions. Deviation of dN/dS (=0.82) to synonymous mutations was obvious at genus rank. At species rank, sequence variability was similar to genus rank, with 283 mutations within *Calycanthus* and 233 within *Chimonanthus* (Table 2). Again the intergenic spacers were more variable than introns and coding regions. The ratios of dN/dS were larger than 1, indicating nonsyonymous mutations had been fixed in the genomes.

### Evolution of structural mutations

#### Indel

There were 178 indels in total (Table 3). Among them 143 (80.3%) were observed at the subfamily rank, 11 (6.2%) at the genus rank, 11 (6.2%) within *Calycanthus*, and 13 (7.3%) within *Chimonanthus*. Most indels occurred in intergenic spacers. Only 10 indels were observed in coding regions (seven in *ycf1*, one in *rpl33*, *rpoC1* and *accD*, respectively). The length of the indels ranged from one to 364 base pairs, but 75.8% of them were not longer than 10 bp. The direction of indel mutations was deletion-biased (Fisher’s exact test, *P* < 0.001). The deletions were about 8.5 times as much as the insertions (Table 3).

**Table 3:** Numbers, directions and locations of indels and repeats in the five chloroplast genomes of Calycanthaceae

		Subfamily	Genus	Species		Total
Mutation type				*Calycanthus*	*Chimonanthus*	
Indel	Total	143	11	11	13	178
Size	1 ~ 10 bp	112	6	5	12	135
	11 ~ 50 bp	19	2	3	1	25
	50 ~ 100 bp	5	3	2	0	10
	>100 bp	7	0	1	0	8
Location	exon	8	1	1	0	10
	intron	23	1	2	1	27
	space	112	9	8	12	141
Direction	insertion	14	1	1	1	17
	deletion	113	10	10	12	145
	uncertain	16	0	0	0	16
Repeat	Total	89	17	7	3	116
Size	3 bp	4	0	0	0	4
	4 bp	25	3	0	0	28
	5 bp	40	9	2	1	52
	6 bp	15	2	2	2	21
	7 bp	1	0	1	0	2
	9 bp	3	0	0	0	3
	10 bp	1	1	0	0	3
	>10 bp	0	2	2	0	4
Location	exon	4	2	0	0	6
	intron	18	2	1	0	21
	space	67	13	6	3	89
Direction	insertion	75	15	6	3	99
	deletion	11	2	1	0	14
	uncertain	3	0	0	0	3

#### Repeat

There were 116 repeating events (Table 3). Among them 89 (76.7%) were observed at the subfamily rank, 17 (14.7%) at the genus rank, 7 (6.0%) within *Calycanthus*, and 3 (2.6%) within *Chimonanthus*. Most repeats occurred in intergenic spacers. Only 6 repeats were observed in coding regions (three in *ycf1*, one in *ndhF*, *rpoC1*, and *rpoC2*, respectively). The length of the repeats ranged from three to 22 base pairs, and four to six base pairs were the most common (84.48%, Fig. 3). Unlike indels, the direction of repeat mutations was insertion-biased (Fisher’s exact test, *P* < 0.001). Approximately 85.3% of the total repeats were insertions.

#### Microsatellite

There were 129 microsatellites, 96 were poly A/T, 13 were poly C/G, eight were 2-bp loci and two were 3-bp loci. All microsatellite loci located in noncoding regions and were A/T-rich indels (Fisher’s exact test, *P* < 0.001). Their mutation directions were not inferable.

#### Inversion

There were 13 inversions of 2-bp to 92-bp in length in the five Calycanthaceae chloroplast genomes (Table 4). All inversions were accompanied by a pair of inverted repeats immediately flanking the inversion (Fig. 2e). Seven inversions occurred at the subfamily rank, two at the genus rank, three at the species rank, and one at both the subfamily and the species ranks. Eight inversions occurred in the LSC region, three in the SSC region, and two in the IR region. A 4-bp inversion occurred within the coding region of *ndhG*, an 8-bp inversion happened in the *ndhA* intron, and 11 inversions were found in intergenic spacers. The franking inverted repeats were from 7 bp to 36 bp in length. No correlation was inferred between the length of the inversions and the inverted repeats (*p* = 0.31). Convergence happened on inversions. For example, both *C. chinensis* and *Ch. nitens* shared an inversion at *trnN*^*GUU*^*-trnR*^*ACG*^, and *C. floridus* and *Ch. praecox* shared the inversion at *psbC-trnS*^*UGA*^ (Table 4).

**Table 4:** The genomic location, length and taxonomic distributions of 13 inversions. uc: direction uncertain; *Cc*: *C. chinensis*; Cf: *C. floridus*; *Chn*: *Ch.nitens*; *Chp*: *Ch. praecox*; *Ia*: *I. australiense*; *Lt*: *Liriodendron tulipifera*; *Mk*: *Magnolia kwangsiensis*

	Location	Length		Inversions							Taxonomic rank
		loop	stem	*Mk*	*Lt*	*Ia*	Cf	*Cc*	*Chn*	*Chp*	
IR	*trnN*^*GUU*^*-trnR*^*ACG*^1	2	14	no	no	no	yes	yes	no	no	Genus
IR	*trnN*^*GUU*^*-trnR*^*ACG*^2	92	17	no	no	no	no	yes	yes	no	Species
LSC	*atpF-atpH*	5	6	uc	uc	no	yes	yes	yes	yes	Subfamily
LSC	*petA-psbJ*	16	36	no	no	yes	no	no	no	no	Subfamily
LSC	*psbC-trnS*^*UGA*^	7	11	no	no	yes	yes	no	no	yes	Species
LSC	*rps18-rpl20*	16	17	no	no	no	yes	yes	yes	yes	Subfamily
LSC	*rps2-rpoC2*	10	9	no	no	yes	no	no	no	no	Subfamily
LSC	*rps4-trnT*	2	7	no	no	no	yes	yes	yes	yes	Subfamily
LSC	*trnS-trnG*	3	12	no	no	no	yes	yes	yes	yes	Subfamily
LSC	*trnT*^*GGU*^*-psbD*	4	7	no	no	yes	yes	yes	no	no	Genus
SSC	*ndhA* intron	8	11	no	yes	yes	no	no	no	no	Subfamily
SSC	*ndhG*	4	9	uc	uc	no	yes	no	no	no	Species
SSC	*rpl32-trnL*	2	8	no	uc	no	yes	yes	no	yes	Subfamily, Species

### Association of structural mutations to nucleotide substitutions

There would exist certain relationships between structural mutation (S) and nucleotide mutations (N). We calculated genetic distances (gd) between regions with and without structural mutations using SNP at three taxonomic ranks (Table 5). It was obvious that the ratio of N to S (N/S) was the smallest at subfamily rank and the largest at species rank. On the contrary, the genetic distances between regions with S (gdS) and without S (gdN) were the largest at subfamily rank and smallest at species rank. Similar patterns existed when only coding or noncoding regions were considered. Noticeably, the gdS was always larger than gdN, suggesting that the nucleotides in the regions with structural mutations were more variable than those in the regions without structural mutations.

**Table 5:** Associations of nucleotide mutations (N) to structural mutations (S). gdN: the genetic distance of the regions without structural mutations; gdS: the genetic distance of the regions with structural mutations. The distances are based on SNPs. *Ia*: *Idiospermum australiense*; *Cc*: *Calycanthus chinensis*; Cf: *Calycanthus floridus*; *Chn*: *Chimonanthus nitens*; *Chp*: *Chimonanthus praecox*

Level	Comparison	Whole genome						Noncoding region			Coding region		
		N	S	N/S	gdS	gdN	gdS/gdN	gdS	gdN	gdS/gdN	gdS	gdN	gdS/gdN
Subfamily	*Ia/Cc*	2664	250	10.66	0.0317	0.0130	2.44	0.0315	0.0145	2.17	0.0323	0.0126	2.56
Subfamily	*Ia/*Cf	2614	249	10.50	0.0312	0.0129	2.42	0.0307	0.0137	2.24	0.0325	0.0127	2.56
Subfamily	*Ia/Chn*	2647	264	10.03	0.0313	0.0129	2.43	0.0307	0.0145	2.12	0.0329	0.0126	2.61
Subfamily	*Ia/Chp*	2587	260	9.95	0.0307	0.0127	2.42	0.0301	0.0137	2.20	0.0325	0.0125	2.60
Genus	*Cc/Chn*	715	46	15.54	0.0076	0.0037	2.05	0.0081	0.0059	1.37	0.0063	0.0032	1.97
Genus	*Cc/Chp*	667	41	16.27	0.0072	0.0033	2.18	0.0077	0.0048	1.60	0.0059	0.0030	1.97
Genus	*Chn/*Cf	687	49	14.02	0.0075	0.0035	2.14	0.0077	0.0049	1.57	0.0070	0.0032	2.19
Genus	*Chp/*Cf	617	44	14.02	0.0069	0.0032	2.16	0.0070	0.0040	1.75	0.0065	0.0030	2.17
Species	*Cc/*Cf	383	17	22.53	0.0043	0.0018	2.39	0.0044	0.0026	1.69	0.0040	0.0016	2.50
Species	*Chp/Chn*	334	16	20.88	0.0033	0.0019	1.74	0.0035	0.0028	1.25	0.0030	0.0017	1.76

### Evolutionary rates of nucleotide and structural mutations

The divergence times of the Calycanthaceae were estimated using a matrix of 83 genes from 27 species of angiosperms (Supplementary Table S3). The total length of the matrix was 63,525 bp. The divergence time of the family was estimated to be 110 Mya from other members in Laurales, 108.8 Mya between the Calycanthoideae and the Idiospermoideae, 18.7 Mya between *Calycanthus* and *Chimonanthus*, 7.6 Mya between the two species of *Calycanthus*, and 6.2 Mya between the two species of *Chimonanthus* (Supplementary Figure S4). The evolutionary rates of the genomes were calculated using the lengths of the genomes, the number of substitutions and the times since divergence. The rates of nucleotide substitution were 0.14 × 10^− 9^ per site per year at the subfamily rank, 0.19 × 10^− 9^ per site per year at the genus rank, 0.34 × 10^− 9^ per site per year in *Calycanthus*, 0.32 × 10^− 9^ per site per year in *Chimonanthus*, and 0.25 × 10^− 9^ per site per year in the Calycanthaceae on average. The mutation rates of indels and repeats were estimated to be 1.73 × 10^− 11^ per site per year. Indels had a rate of 0.64 × 10^− 11^ per site per year and repeats had a rate of 1.0 × 10^− 11^ per site per year. The rate of microsatellites and the inversions were not considered because these mutations lacked phylogenetic information and the directions were not inferable.

## Discussion

Mutations are at least the raw materials if not a drive in evolution [5]. It is tedious to count the molecular mutations even in the small genomes such as chloroplast genomes of plants and most of our understanding of evolution is based on small regions instead of the whole genome. What seems more difficult is to determine the directions of mutations, a critical concept in evolution. The inherently obvious phylogenetic relationships within Calycanthaceae serve as an ideal reference for studying the chloroplast genome evolution at subfamily, genus and species ranks.

### General patterns of chloroplast genome evolution

Mutations in Calycanthaceae had experienced a long history of natural selection and exhibited some unique patterns that changed our general notion on genome evolution. It is believed that there are sites which mutate more frequently than other sites. Such kind of sites were very rare in Calycanthaceae and perhaps in other species as well. About 98% of single nucleotide mutant sites had two alleles and only 22 (0.79%) sites had more than two alleles. Reverse mutations should be even rarer (2.03%). This implies that modeling the evolution of such uncertain sites does not add much knowledge to the study.

Nucleotide mutations are of unequal possibilities. Changes from C or G to A or T were more common in the chloroplast genomes of Calycanthaceae (and other seed plants) [12, 13]. This explains why A or T is about 10% more than G or C. However, such kind of biased mutations happened only in the noncoding regions [12, 44]. The mutations biased to the opposite side in the coding regions. The G and C content of the coding genes was approximately 60%. Of course there are a few exceptions, esp. non-seed plants, for example, *Selaginella* [45]. It is postulated that the mutation rate of noncoding regions is higher than that of the coding regions. In our case, it is true for intergenic spacer regions. The nucleotide mutation rate in introns was almost the same as that of coding regions. Synonymous substitutions which do not change the function of a gene, are believed to be more frequently present than nonsynonymous substitutions. However, in the coding genes of the chloroplast genomes of Calycanthaceae, dN/dS equaled one, indicating a balanced synonymous and nonsynonymous mutations.

There are mutation peaks and valleys in the plastid genomes owing to functional constrains [46], and nonsynonymous mutations are believed to be a genetic force that generates heterogeneity [47]. The dN/dS, the ratio indicating natural selection pressure, varies across the plastid genomes of the Calycanthaceae. Photosynthetic apparatus genes have the lowest ratio of dN/dS, but the *rpo-*genes show relatively high dN/dS (Fig. 4). Therefore, “lucky genes” might sometimes be found even from coding genes for phylogeny [48].

Structural mutations are not as common as nucleotide substitutions. Structural mutations occur mostly in noncoding regions, especially for microsatellite mutations (Table 2 and Table 3). Motifs of a single nucleotide repeats are much more common than the typical motifs of two to six nucleotides [14, 49, 50]. Structural mutations are characterized by insertions, deletions, repeats, inversions and microsatellites of small length, usually shorter than 10 bases or base pairs [14].

### Direction of mutations

Although there are eight possible transversional changes and only four transitional changes, transitions are much more common in the chloroplast genomes. Among the eight transversions, their frequencies are different. Mutations of A → C + T → G and C → A + G → T were much more common than other mutations in the chloroplast genomes of Calycanthaceae. There is a nucleotide T bias in chloroplast genomes. Methylation-deamination and uncorrected post-replicative transition mutation lead to the replacement of C with T. The same phenomenon was observed on nuclear genome data [21, 51] and animal mitochondrial genome data [52].

Structural mutations are potential informative phylogenetic characters of low homoplasy if handled properly. For example, *Arachis* [53], yam [54], and *Cynara* [55] have 85, 43, 34 indels in their whole chloroplast genomes, respectively. However, the direction of structural mutations are of less focus [56]. Same indels and repeats do not occur by chance in different taxa and data of correctly coded indels and repeats are of very low homoplasy in phylogeny (Table 3). In Calycanthacee the direction of indels and repeats were easily inferable. For indels, deletions were much more common than insertions; and for repeats, repeat gains were much more than repeat losses. Microsatellites are sometimes used in phylogenetic analyses and a repeat number increase was observed in maize short term evolution [52]. However, in this study the directions of microsatellite mutations in the chloroplast genomes of Calycanthaceae did not have a general tendency either to increase or to decrease at family, genus or species ranks. Therefore, inclusion of the gaps induced by microsatellites above species rank is likely to introduce homoplasy into datasets. Parallel mutations of inversions among individuals were observed in Calycanthaceae. Inversions often happen due to repetitive structures by parallel or back mutations during chloroplast genome evolution [17, 19]. Inversions must be identified when doing alignment but the gaps induced by inversions should be used with caution above species rank. In short, the structural mutations are often a mixture of phylogenetic signals and noises and only phylogenetic informative structural mutation information is considered when nucleotide data are insufficient to resolve a phylogeny [57, 58].

### Taxonomic rank- and genome position-related mutations

It is now a consensus that mutation rate varies among lineages [59]. Many studies showed that annual plants evolve faster than perennials and trees [5, 60]. Our study demonstrated an accelerated evolution from subfamily to species in Calycanthaceae. The mutation rate is the lowest at subfamily rank and highest at species rank. Such a heterogeneity of mutation rates among taxonomic ranks is probably due to life history, time of selection and random mutations [5, 61]. In Calycanthaceae, the ancestors were likely to be large trees of very long life history such as *I. australiense*. Shrubs of relatively short life history are a derived character state [60]. From the branch lengths it is easy to understand that ancestors of higher taxonomic ranks experienced longer natural selection that might have eliminated quite many variations. Evolution might accelerate in one historical period and decelerate in the other.

Although it is well known that mutation rates vary significantly among genes of genomes [62]. Some regions have been proven more variable than others [63–65], for example, *ycf1*, *trnH-psbA*, *rpl32-trnL*. Generally ribosomal protein-related and photosynthetic metabolic genes had higher mutation rates than photosynthetic apparatus genes in Calycanthaceae chloroplast genome (Fig. 4). There are two explanations. The first one is that the ribosomal protein-related and photosynthetic metabolic genes have relaxed evolutionary constraint. For example, the *rpl32* have been completely lost from the chloroplast genome in multiple lineages within the land plants [66, 67]. The second one is the functions of some of these genes have been taken over by some genes in the nuclear genome and strong coevolution between plastid- and nuclear-encoded subunits that have accelerated mutation rates [67–70]. The regions of rich structural mutations also had higher nucleotide diversity in the chloroplast genomes [44, 71, 72].

## Conclusions

A detailed understanding of the characteristics of the chloroplast genome evolution is helpful for correct use of chloroplast genome data in evolutionary biology. We observed the taxonomic and genomic distributions of mutations existed in the five chloroplast genomes of Calycanthaceae, inferred their directions, and estimated the rate of evolution. These direct observations provide raw data for computer simulation and modeling true evolution of sequence data.

## Supplementary information

**Additional file 1: Table S1**. Sample accession numbers of the four species in Calycanthaceae analyzed in this study and their localities with voucher information.

**Additional file 2: Table S2.** List of specific primers used to amplify and sequence the chloroplast genomes of Calycanthaceae.

**Additional file 3: Table S3.** List of taxa used for estimating the divergence time of the Calycanthaceae.

**Additional file 4: Table S4**. List of genes found in the chloroplast genomes of Calycanthaceae.

**Additional file 5: Figure S1.** Chloroplast genome map of *Chimonanthus praecox*, a representative of all four newly determined chloroplast genomes. The genes inside and outside the circle are transcribed clockwise and counterclockwise, respectively. Genes that belong to different functional groups are represented by different colors. The thick lines indicate the extent of the inverted repeats (IRa and IRb) that separate the genomes into small (SSC) and large (LSC) single-copy regions. The flower in the center is of *Chimonanthus praecox.*

**Additional file 6: Figure S2**. Detailed view of the border regions between the inverted repeats and the single-copy regions of the Calycanthaceae chloroplast genomes. The figure is not to scale.

**Additional file 7: Figure S3.** The codon usage in the Calycanthaceae chloroplast genomes.

**Additional file 8: Figure S4.** Divergence times of the crown groups estimated using BEAST version 1.6.1 under the uncorrelated lognormal (UCLN) model based on 83 chloroplast genes of 26 taxa. Numbers on the nodes are the estimated medium ages (Mya).

## Data Availability

The datasets supporting the results of this article are included within additional files. All nucleotide sequences were deposited in the NCBI GenBank repository (MH377056 - MH377059).

## Abbreviations

MA: Mutation accumulation
Mya: Million years ago
CTAB: Cetyl trimethylammonium bromide
DOGMA: Dual organellar genome annotator
PCR: Polymerase chain reaction
IR: Inverted repeat
LSC: Large single copy
SSC: Small single copy
dN: Number of substitutions per nonsynonymous site
dS: Number of substitutions per synonymous site
