# Supplementary material for: Evolutionary directions of single nucleotide substitutions and structural mutations in the chloroplast genomes of the family Calycanthaceae
Source: BMC Evol Biol. 2020 Jul 31;20:96. doi: 10.1186/s12862-020-01661-0 (PMC7393888; doi:10.1186/s12862-020-01661-0)
Supplement: Supplementary file 1 — Additional file 1: Table S1. Sample accession numbers of the four species in Calycanthaceae analyzed in this study and their localities with voucher information. [file 12862_2020_1661_MOESM1_ESM.docx]

**Table S1**. Accession numbers of the four Calycanthaceae used in this study and their localities with voucher information.

| Taxon | Locality | Voucher |
| --- | --- | --- |
| *Calycanthus chinensis* Cheng et S. Y. Chang | Lin’an, Zhejiang, China | S. L. Zhou 2000615 (PE) |
| *Chimonanthus nitens* Oliver | Guilin,Guangxi, China | Zhou Shiliang(PE) |
| *Chimonanthus praecox* (L.) Link | Beijing Bot. Gard., Beijing, China | S.L. Zhou 0020 (PE) |
| *Idiospermum australiense* (Diels) S.T. Blake | New York Bot. Gard., New York, USA | Wen s. n. (F) |
