# Supplementary material for: Evolutionary directions of single nucleotide substitutions and structural mutations in the chloroplast genomes of the family Calycanthaceae
Source: BMC Evol Biol. 2020 Jul 31;20:96. doi: 10.1186/s12862-020-01661-0 (PMC7393888; doi:10.1186/s12862-020-01661-0)
Supplement: Supplementary file 2 — Additional file 2: Table S2. List of specific primers used to amplify and sequence the chloroplast genomes of Calycanthaceae. [file 12862_2020_1661_MOESM2_ESM.docx]

**Table S2**. List of specific primers used to amplify and sequence the chloroplast genomes of Calycanthaceae.

| Name | Forward primer | Name | Reverse primer | Length (bp) |
| --- | --- | --- | --- | --- |
| Caly_2kF | AAGTTGACAAGGTGATATTT | Caly_2kR | CTACGCACTTGCTCATGATCAT | 833 |
| Caly_39kF | TATACTTCGACCAGCATTGAATG | Caly_39kR | GGTGAATATCGCTTATTCCGG | 1150 |
| Caly_64kF | CGGACATCTATTTCAAACGCA | Caly_64kR | TAGATACAAAAAACTATCGATG | 1168 |
| Caly_83kF | TTATCTAGACGAACCCGGAACAT | Caly_83kR | ATACGAACTCAATTCATTATCG | 1300 |
| Caly_95kF | TATTCAATCACATAGTTTGGGC | Caly_95kR | TTAGAATTAGGCTCGGTCAACT | 1131 |
| Caly_113kF | TGATCATGAGACATATAATTG | Caly_113kR | CAATCAATCGGTTCCACAATGAA | 833 |
| Caly_122kF | TGATAGGAACGAACAGGAACA | Caly_122kR | GTCGAAACTGTTTACCCCAAGA | 1303 |
