# Supplementary material for: Evolutionary directions of single nucleotide substitutions and structural mutations in the chloroplast genomes of the family Calycanthaceae
Source: BMC Evol Biol. 2020 Jul 31;20:96. doi: 10.1186/s12862-020-01661-0 (PMC7393888; doi:10.1186/s12862-020-01661-0)
Supplement: Supplementary file 3 — Additional file 3: Table S3. List of taxa used for estimating the divergence time of the Calycanthaceae. [file 12862_2020_1661_MOESM3_ESM.docx]

**Table S3.** List of taxa used for estimating the divergence time of the Calycanthaceae.

|  | **Taxon** | **Species** | **GenBank accession number** |
| --- | --- | --- | --- |
| 1 | Brassicaceae | *Arabidopsis thaliana* | NC_000932 |
| 2 | Rosaceae | *Prunus persica* | NC_014697 |
| 3 | Penthoraceae | *Penthorum chinense* | JX436155 |
| 4 | Asteraceae | *Helianthus annuus* | NC_007977 |
| 5 | Solanaceae | *Nicotiana tabacum* | NC_001879 |
| 6 | Polygonaceae | *Fagopyrum esculentum* subsp. *ancestrale* | NC_010776 |
| 7 | Buxaceae | *Buxus microphylla* | NC_009599 |
| 8 | Nelumbonaceae | *Nelumbo lutea* | JQ336992 |
| 9 | Ranunculaceae | *Ranunculus macranthus* | NC_008796 |
| 10 | Ceratophyllaceae | *Ceratophyllum demersum* | NC_009962 |
| 11 | Typhaceae | *Typha latifolia* | NC_013823 |
| 12 | Acoraceae | *Acorus americanus* | NC_010093 |
| 13 | Magnoliaceae | *Liriodendron tulipifera* | NC_008326 |
| 14 | Magnoliaceae | *Magnolia kwangsiensis* | NC_015892 |
| 15 | Piperaceae | *Piper cenocladum* | NC_008457 |
| 16 | Winteraceae | *Drimys granadensis* | NC_008456 |
| 17 | Chloranthaceae | *Chloranthus spicatus* | NC_009598 |
| 18 | Illiciaceae | *Illicium oligandrum* | NC_009600 |
| 19 | Nymphaeaceae | *Nymphaea alba* | NC_006050 |
| 20 | Nymphaeaceae | *Nuphar advena* | NC_008788 |
| 21 | Amborellaceae | *Amborella trichopoda* | NC_005086 |
