# Supplementary figures and images for: Evolutionary directions of single nucleotide substitutions and structural mutations in the chloroplast genomes of the family Calycanthaceae
Source: BMC Evol Biol. 2020 Jul 31;20:96. doi: 10.1186/s12862-020-01661-0 (PMC7393888; doi:10.1186/s12862-020-01661-0)

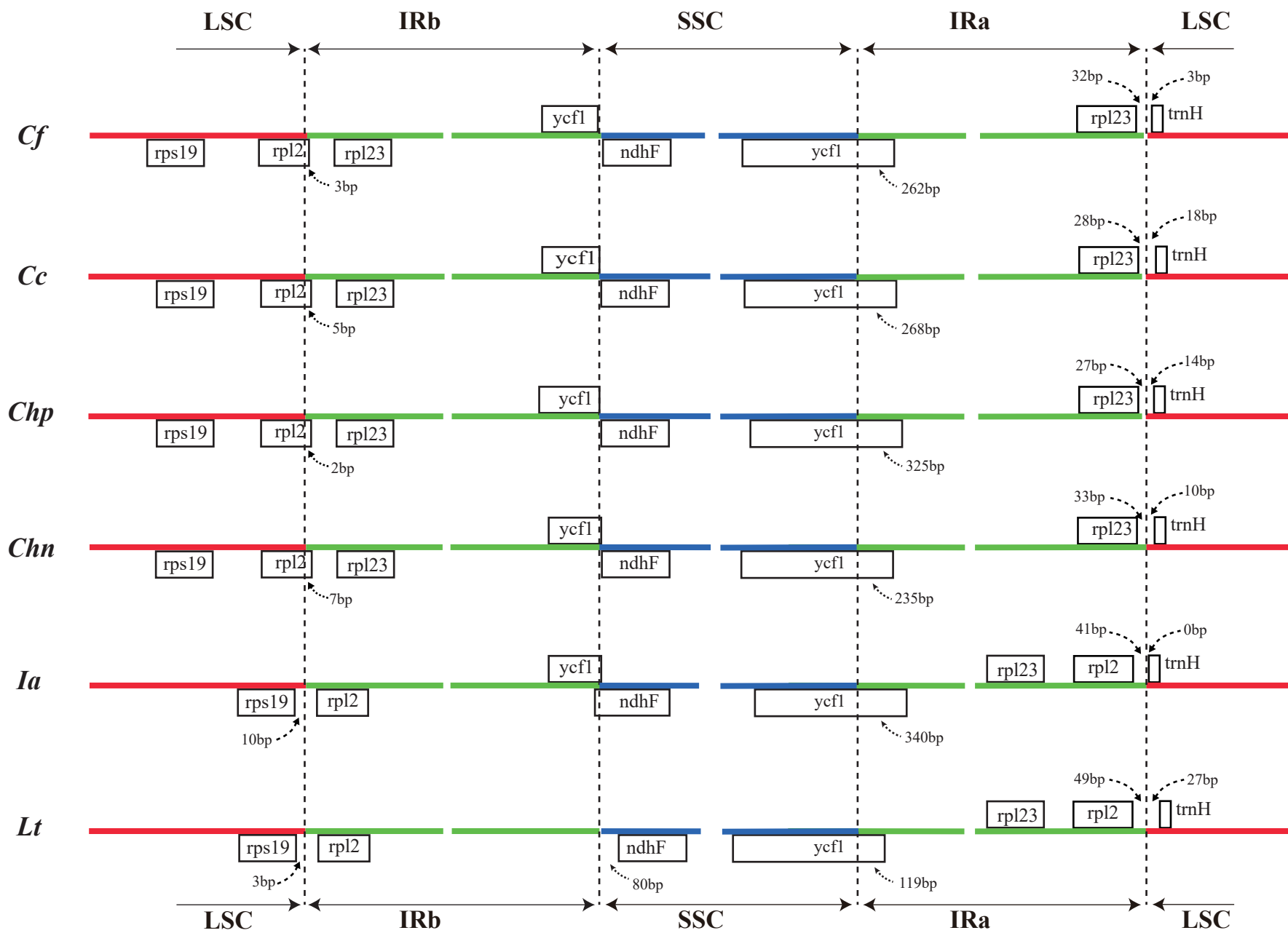

Supplement: Supplementary file 6 — Additional file 6: Figure S2. Detailed view of the border regions between the inverted repeats and the single-copy regions of the Calycanthaceae chloroplast genomes. The figure is not to scale. [file 12862_2020_1661_MOESM6_ESM.pdf]

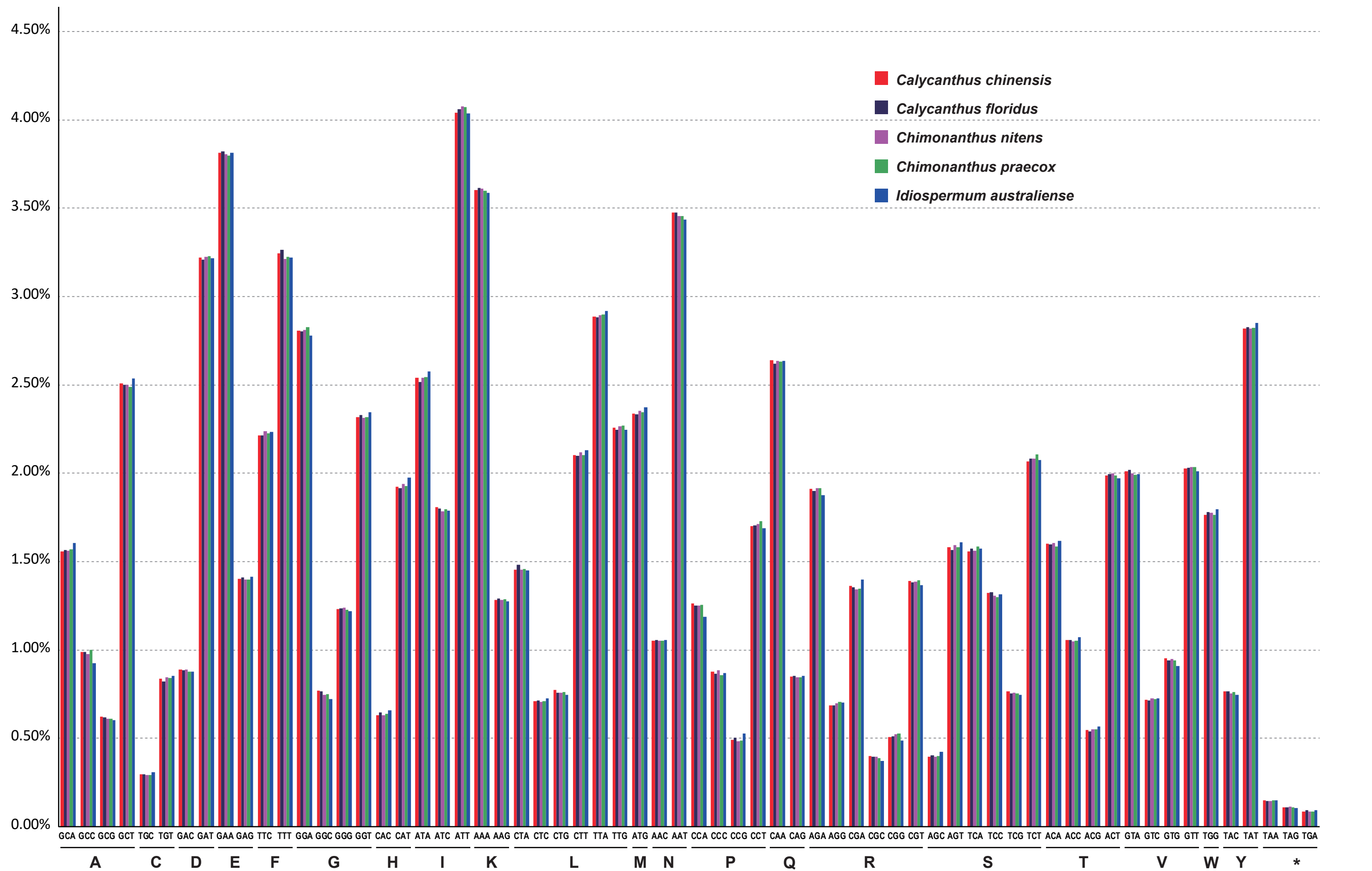

Supplement: Supplementary file 7 — Additional file 7: Figure S3. The codon usage in the Calycanthaceae chloroplast genomes. [file 12862_2020_1661_MOESM7_ESM.pdf]

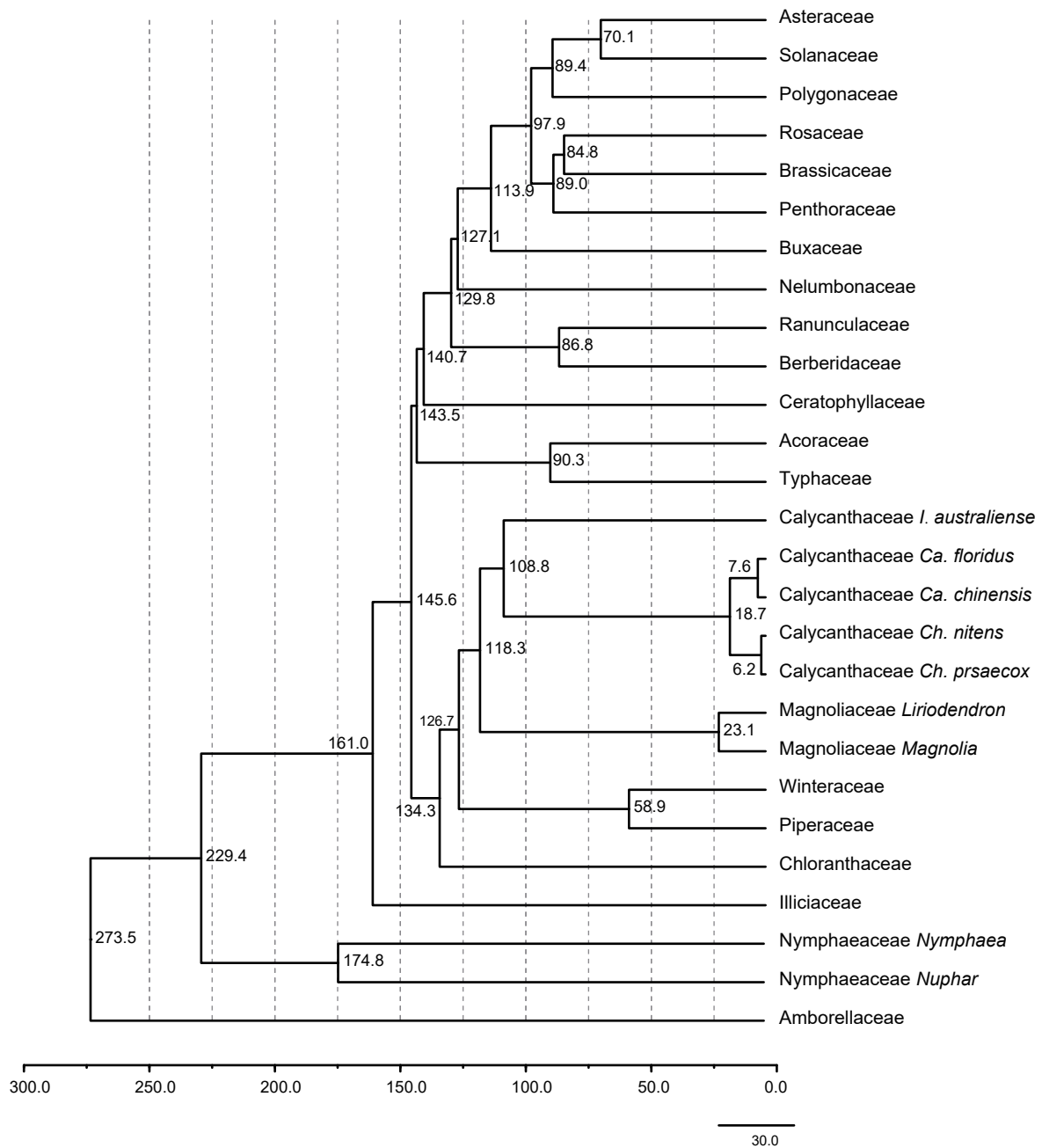

Supplement: Supplementary file 8 — Additional file 8: Figure S4. Divergence times of the crown groups estimated using BEAST version 1.6.1 under the uncorrelated lognormal (UCLN) model based on 83 chloroplast genes of 26 taxa. Numbers on the nodes are the estimated medium ages (Mya). [file 12862_2020_1661_MOESM8_ESM.pdf]
