# Supplementary material for: Evolutionary directions of single nucleotide substitutions and structural mutations in the chloroplast genomes of the family Calycanthaceae
Source: BMC Evol Biol. 2020 Jul 31;20:96. doi: 10.1186/s12862-020-01661-0 (PMC7393888; doi:10.1186/s12862-020-01661-0)
Supplement: Supplementary file 5 — Additional file 5: Figure S1. Chloroplast genome map of Chimonanthus praecox, a representative of all four newly determined chloroplast genomes. The genes inside and outside the circle are transcribed clockwise and counterclockwise, respectively. Genes that belong to different functional groups are represented by different colors. The thick lines indicate the extent of the inverted repeats (IRa and IRb) that separate the genomes into small (SSC) and large (LSC) single-copy regions. The flower in the center is of Chimonanthus praecox. [file 12862_2020_1661_MOESM5_ESM.pdf]

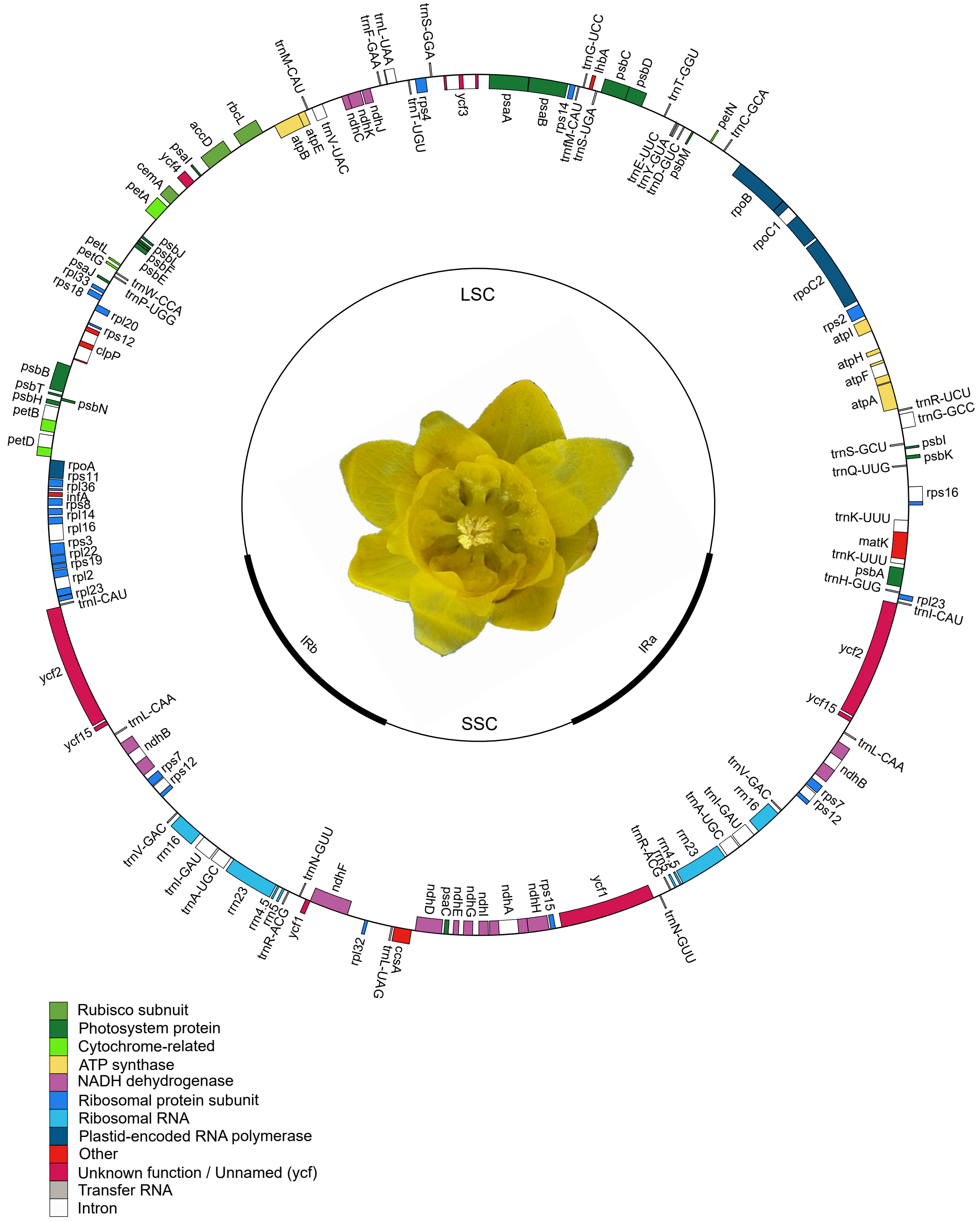

LSC
SSC
IRa
IRb
trnM-CAU
trnL-UAA
trnF-GAA
trnS-GGA
trnG-UCC
lhbA
psbC
psbD
trnT-GGU
petN
trnC-GCA
rbcL
accD
psaI
ycf4
cemA
petA
atpE
atpB
trnV-UAC
ndhJ
ndhK
ndhC
rps4
trnT-UGU
ycf3
psaA
psaB
rps14
trnfM-CAU
trnS-UGA
trnE-UUC
trnY-GUA
trnD-GUC
psbM
rpoB
rpoC1
rpoC2
rps2
atpI
atpH
atpF
atpA
trnR-UCU
trnG-GCC
psbI
psbK
trnS-GCU
trnQ-UUG
rps16
trnK-UUU
matK
trnK-UUU
psbA
trnH-GUG
rpl23
trnI-CAU
ycf2
ycf15
trnL-CAA
ndhB
rps7
rps12
trnV-GAC
rrn16
trnI-GAU
trnA-UGC
rrn23
rrn4.5
rrn5
trnR-ACG
trnN-GUU
ycf1
rps15
ndhH
ndhA
ndhI
ndhG
ndhE
psaC
ndhD
ccsA
trnL-UAG
rpl32
ndhF
psbJ
psbL
psbF
psbE
petL
petG
psaJ
rpl33
rps18
trnW-CCA
trnP-UGG
rpl20
rps12
clpP
psbB
psbT
psbH
petB
psbN
petD
rpoA
rps11
rpl36
infA
rps8
rpl14
rpl16
rps3
rpl22
rps19
rpl2
rpl23
trnI-CAU
ycf2
ycf15
trnL-CAA
ndhB
rps7
rps12
trnV-GAC
rrn16
trnI-GAU
trnA-UGC
rrn23
rrn4.5
rrn5
trnR-ACG
ycf1
trnN-GUU
ndhF
Rubisco subunit
Photosystem protein
Cytochrome-related
ATP synthase
NADH dehydrogenase
Ribosomal protein subunit
Ribosomal RNA
Plastid-encoded RNA polymerase
Other
Unknown function / Unnamed (ycf)
Transfer RNA
Intron
